# Inhibition of Autophagic Degradation Process Contributes to Claudin-2 Expression Increase and Epithelial Tight Junction Dysfunction in TNF-α Treated Cell Monolayers

**DOI:** 10.3390/ijms18010157

**Published:** 2017-01-17

**Authors:** Cong Zhang, Junkai Yan, Yongtao Xiao, Yujie Shen, Jiazheng Wang, Wensong Ge, Yingwei Chen

**Affiliations:** 1 Department of Gastroenterology, Xin Hua Hospital Affiliated to Shanghai Jiao Tong University School of Medicine, Shanghai 200092, China; zhang-cong@sjtu.edu.cn (C.Z.); shenyujie@sjtu.edu.cn (Y.S.); wangjiazheng1991@163.com (J.W.); 2 Shanghai Key Laboratory of Pediatric Gastroenterology and Nutrition, Shanghai Institute for Pediatric Research, Shanghai 200092, China; yanjunkai2015@sjtu.edu.cn (J.Y.); ytxiaocell@163.com (Y.X.)

**Keywords:** claudin-2, barrier function, autophagy, inflammatory, TNF-α

## Abstract

Tight junction dysfunction plays a vital role in some chronic inflammatory diseases. Pro-inflammatory cytokines, especially tumor necrosis factor alpha (TNF-α), act as important factors in intestinal epithelial tight junction dysfunction during inflammatory conditions. Autophagy has also been shown to be crucial in tight junction function and claudin-2 expression, but whether autophagy has an effect on the change of claudin-2 expression and tight junction function induced by TNF-α is still unknown. To answer this question, we examined the expression of claudin-2 protein, transepithelial electrical resistance (TER), and permeability of cell monolayers, autophagy flux change, and lysosomal pH after TNF-α with or without PP242 treatment. Our study showed that claudin-2 expression, intestinal permeability, microtubule-associated protein 1 light chain 3B II (LC3B-II) and sequestosome 1 (P62) expression largely increased while TER values decreased in TNF-α treated cell monolayers. Further research using 3-methyladenine (3-MA), bafilomycin A1, and ad-mCherry-GFP-LC3B adenovirus demonstrated that LC3B-II increase induced by TNF-α was attributed to the inhibition of autophagic degradation. Moreover, both qualitative and quantitative method confirmed the increase of lysosomal pH, and mammalian target of rapamycin (mTOR) inhibitor PP242 treatment relieved this elevation. Moreover, PP242 treatment also alleviated the change of autophagy flux, TER, and claudin-2 expression induced by TNF-α. Therefore, we conclude that increase of claudin-2 levels and intestinal epithelial tight junction dysfunction are partly caused by the inhibition of autophagic degradation in TNF-α treated cell monolayers.

## 1. Introduction

Intestinal epithelial physical barrier plays an important role in protecting organisms from harmful substances in lumen [1]. This mostly depends on intercellular tight junctions (TJs), which have shown to be responsible for material transport and maintaining barrier function [2]. Unlike the function of most TJs, the expression of claudin-2 protein increases paracellular permeability and is inversely associated with transepithelial electrical resistance (TER) [3,4]. Some studies have demonstrated that inflammatory diseases, such as inflammatory bowel disease (IBD) and necrotizing enterocolitis (NEC), are associated with increased expression of claudin-2 and intestinal barrier defect [5,6]. Moreover, further research also confirmed that inflammatory cytokines (TNF-α, interferon-γ and interleukins etc.) are crucial in this process [7,8]. Therefore, studies designed to figure out mechanisms of the change of tight junction function in inflammatory conditions would promote our understanding for the etiology of these diseases.

Autophagy is a highly conserved degradation process that removes damaged or aged proteins and organelles to maintain homeostasis in eukaryotic cells. During stress conditions (starvation, hypoxia etc.), autophagy is initiated, and subsequently an isolation membrane involved cytoplasmic constituents comes to being a double-membrane structure (autophagosome), which then fuses with lysosomes to form an autophagolysosome for the degradation of the inner membrane and cargo [9]. It has been shown that autophagy takes part in many biological processes, such as cell death and development [10,11]. In addition, recent study has demonstrated that autophagy increases TJs barrier function through claudin-2 protein degradation in starvation conditions [12]. Considering that autophagy is a dynamical process that changes with different environments [13,14], we speculate that autophagy may play a role in claudin-2 levels and intestinal tight junction function during inflammatory conditions. To validate this hypothesis, we used TNF-α treated an intestinal epithelial model system to simulate inflammatory condition in vitro, then we analyzed the change of TER, intestinal permeability, lysosomal pH, claudin-2, LC3B-II, and P62 protein. Furthermore, we have investigated the role of lysosomal acidic environment in these changes. Our data showed that claudin-2 levels and intestinal epithelial tight junction function markedly changed in TNF-α treated cell monolayers, which could be resulted by the inhibition of autophagic degradation process.

## 2. Results

### 2.1. The Claudin-2, LC3B-II, and P62 Expression Increased in TNF-α Treated Caco-2 Cell Monolayers

**Figure 1:**
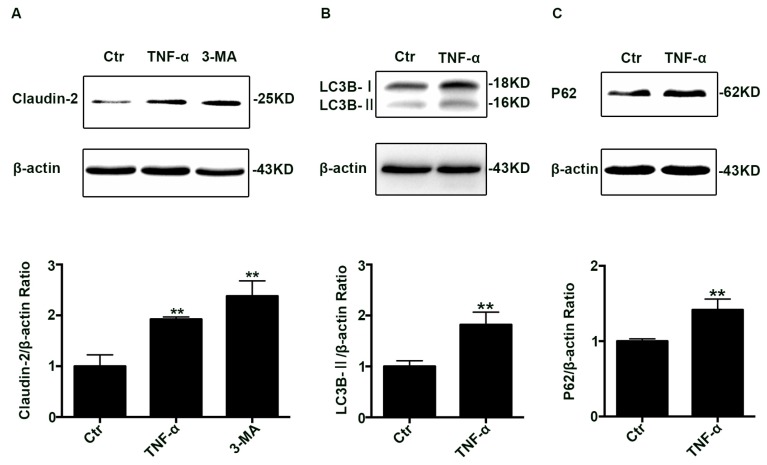
Change of claudin-2, LC3B-II, and P62 protein in TNF-α treated Caco-2 cell monolayers. (**A**) Western blotting analysis of caludin-2 in Caco-2 cell monolayer after TNF-α (10 ng/mL) administration or 3-MA (5 mM) treatment for 48 h. (**B**,**C**) Western blotting analysis of LC3B-II and P62 in Caco-2 cells after TNF-α (10 ng/mL) administration for 48 h. β-actin was used as an internal control. Data were shown as mean ± SD and replicated three times. ** *p* < 0.01 versus control group. Ctr, control group.

To investigate claudin-2 levels in inflammatory conditions, differentiated Caco-2 cell monolayers were treated with TNF-α (10 ng/mL) for 48 h. Using western blotting analysis, we found that claudin-2 protein levels increased (Figure 1A). In addition, we also studied the effect of the autophagy inhibitor 3-MA on claudin-2 expression. The western blotting analysis showed that 3-MA increased the claudin-2 level (Figure 1A). Taken together, these data demonstrated that claudin-2 expression increased after TNF-α administration or 3-MA treatment.

Considering that the trend of claudin-2 levels treated by TNF-α was the same as 3-MA, then we explored the status of autophagy in TNF-α induced claudin-2 increase. To evaluate autophagy, we detected the level of LC3B-II and P62 proteins, and the western blotting analysis showed that both the expression of LC3B-II (Figure 1B) and P62 (Figure 1C) increased at the same time. Commonly, LC3B-II is interpreted as a marker of autophagy induction, and P62 protein is degraded mainly through autophagy-lysosome pathway. However, it is important to point out that the increase of LC3B-II existed not only when the autophagy flux was increased, but also when the autophagic degradation was inhibited.

### 2.2. Autophagic Degradation Was Inhibited in TNF-α Treated Caco-2 Cells

**Figure 2:**
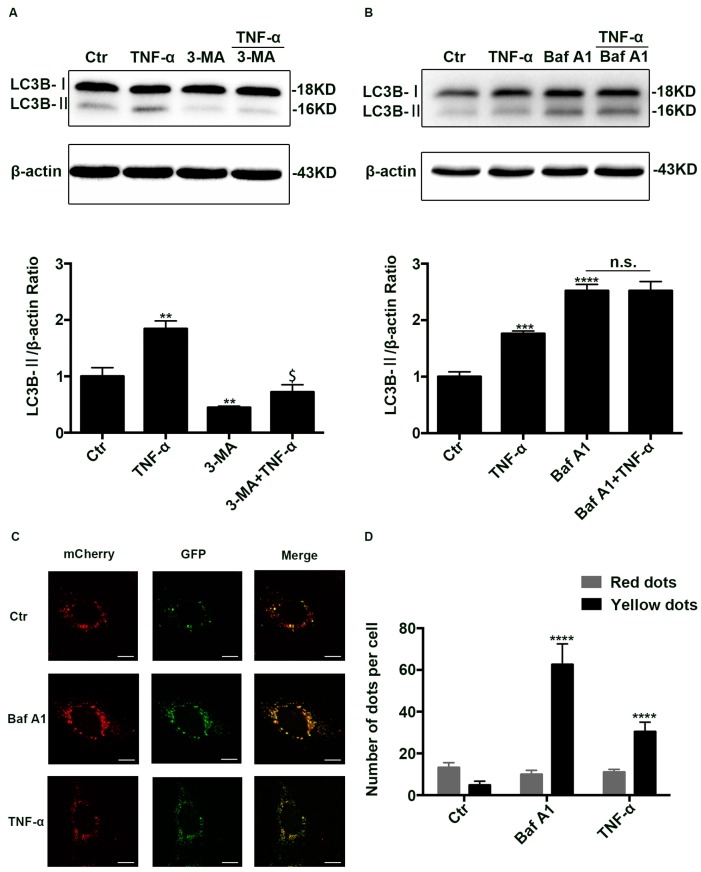
The inhibition of autophagic degradation in TNF-α treated cells. (**A**,**B**) Western blotting analysis of LC3B-II in Caco-2 cells treated with TNF-α (10 ng/mL) in the presence or absence of 3-MA (5 mM, 24 h) or Baf A1 (100 nM, 4 h) pretreatment. (**C**) Representative images of Caco-2 cells transfected with ad-mCherry-GFP-LC3B adenovirus after TNF-α (10 ng/mL, 48 h) or Baf A1 treatment (100 nM, 4 h). Scale bar: 15 μm. (**D**) The number of red and yellow LC3 dots per cell were counted under confocal microscope (>30 cells/group). Data were shown as mean ± SD and replicated three times. ** *p* < 0.01, *** *p* < 0.001, **** *p* < 0.0001 versus control group. $ *p* < 0.05 versus 3-MA group. n.s., no significant. Ctr, control group; 3-MA, 3-methyladenine; Baf A1, Bafilomycin A1.

To figure out the reason of LC3B-II increase induced by TNF-α, autophagy inhibitor 3-MA (5 mM) and autophagic degradation inhibitor Bafilomycin A1 (100 nM) was used before TNF-α treatment. As reported previously, 3-MA decreased the levels of LC3B-II (Figure 2A) while bafilomycin A1 enhanced it (Figure 2B) [15,16]. Nevertheless, TNF-α was still enhanced the amount of LC3B-II though pretreated with 3-MA (Figure 2A), but could not further promote the expression of LC3B-II even when pretreated with bafilomycin A1 (Figure 2B), implying autophagic degradation was inhibited.

To further clarify the mechanism, Caco-2 cells were transfected with Ad-mCherry-GFP-LC3B adenovirus. Owing to the fact that green fluorescence was quenched in the acid environment of lysosome, we can only observe yellow and red dots. Yellow dots (merged by mCherry and GFP fluorescence) indicate the autophagosomes that are not fused with lysosome, while red dots (mCherry fluorescense) indicate the compartments that have been fused with lysosome. Thus, the increase of both red and yellow dots indicates the activation of autophagy, while the increase of yellow dots alone means that the degradation process of autophagy is inhibited. As reported previously, bafilomycin A1 treatment, which inhibited autophagosome-lysosome fusion, only markedly increased the number of yellow dots. Similarly, TNF-α treatment largely enhanced the yellow dots while it did not increase the number of red dots (Figure 2C,D), implying autophagic degradation was blocked. To sum up, all those results indicate that the degradation process of autophagy-lysosome pathway is blocked after TNF-α administration.

### 2.3. Lysosomal Acidic Environment Was Compromised and PP242 Rescued It in TNF-α Treated Caco-2 Cells

**Figure 3:**
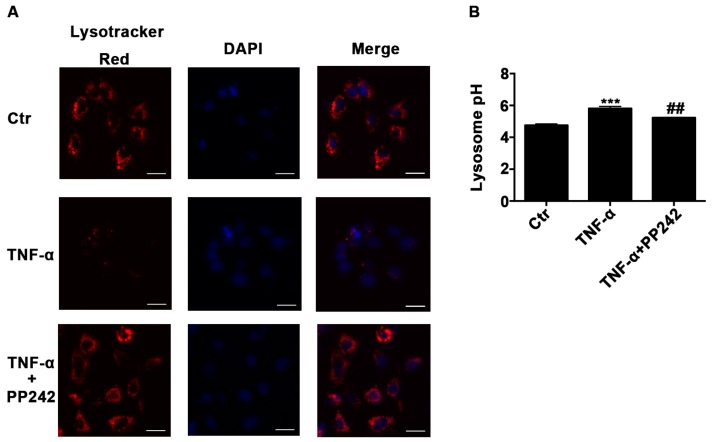
Lysosomal acidic environment was impaired after TNF-α treatment and PP242 rescued it. (**A**) Images of Caco-2 cells labeled with Lysotracker Red DND-99 dye after TNF-α (10 ng/mL, 48 h) and/or PP242 (1 μm, 24 h) treatment. The red and blue fluorescence were represented the lysosome and nucleus, respectively. Scale bar: 30 μm. (**B**) Lysosomal pH changes of Caco-2 cells labeled with Lysosensor Yellow/Blue DND-160 after TNF-α and/or PP242 treatment. Data were shown as mean ± SD and replicated three times. *** *p* < 0.001 versus control group. ## *p* < 0.01 versus TNF-α group. Ctr, control group.

It has been shown that an acidic environment is important for the activation of lysosomal proteases in autophagic degradation process [17]. To study the role of lysosomal pH in the impaired autophagy flux induced by TNF-α, we used Lysotracker Red DND-99 and Lysosensor Yellow/Blue DND-160 to measure the pH of lysosome qualitatively and quantitatively. We observed that red fluorescence dramatically decreased after TNF-α treatment (Figure 3A), indicating an increase of lysosomal pH, and the quantitative method confirmed that TNF-α increased the pH value from 4.78 ± 0.07 to 5.81 ± 0.11 (Figure 3B). Then mTOR inhibitor PP242, which has been shown to promote lysosomal biogenesis [18], was applied after TNF-α treatment for 24 h. As expected, PP242 enhanced the red fluorescence and rescued the pH value increase induced by TNF-α from 5.81 ± 0.11 to 5.23 ± 0.02 (Figure 3B). Therefore, the acidic condition of lysosome was compromised after TNF-α administration and PP242 relieved this effect.

### 2.4. PP242 Relieved the Change of Autophagy Flux Induced by TNF-α

**Figure 4:**
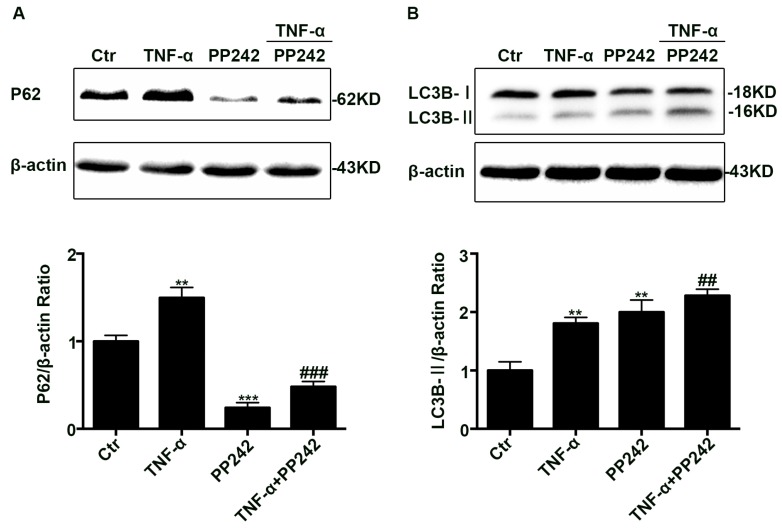
PP242 alleviated the change of autophagy flux caused by TNF-α. (**A**,**B**) Western blot analysis of P62 and LC3B-II after TNF-α (10 ng/mL, 48 h) and/or PP242 (1 μm, 24 h) treatment. Data were shown as mean ± SD and replicated three times. ** *p* < 0.01, *** *p* < 0.001 versus control group. ## *p* < 0.01, ### *p* < 0.001 versus TNF-α group. Ctr, control group.

To verify whether PP242 also has impact on the impairment of autophagy flux induced by TNF-α, Caco-2 cell monolayers were administrated with PP242 after TNF-α treatment. Using western blotting assay, we found that PP242 administration relieved P62 increase induced by TNF-α (Figure 4A), but enhanced LC3B-II levels more compared with TNF-α group (Figure 4B). As we all know, PP242 is an inducer of autophagy, so it could further increase the LC3B-II levels after TNF-α treatment. Considering that P62 is degraded by autophagolysosome, this decrease of P62 supports PP242 in alleviating the disrupted autophagy flux leaded by TNF-α.

### 2.5. PP242 Partly Relieved the Change of Claudin-2 Expression and Intestinal Epithelial Tight Junction Function Induced by TNF-α

**Figure 5:**
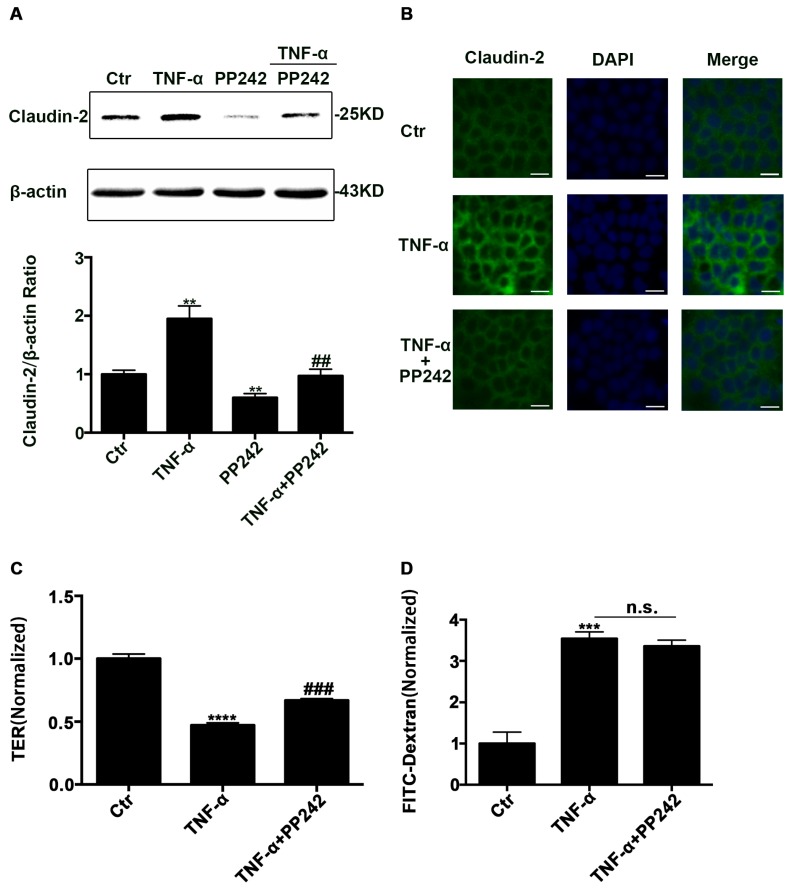
PP242 alleviated the change of claudin-2 expression and intestinal epithelial tight junction function in TNF-α treated Caco-2 cells. (**A**) Western blot analysis of claudin-2 after TNF-α (10 ng/mL, 48 h) and/or PP242 (1 μm, 24 h) treatment. (**B**) Immunofluorescence staining of claudin-2 after TNF-α and/or PP242 treatment in Caco-2 cell monolayers. The green and blue fluorescence were represented the claudin-2 protein and nucleus, respectively. Scale bar: 20 μm. (**C**) The effect of PP242 (1 μm, 24 h) on TER of Caco-2 cell monolayers treated with TNF-α (10 ng/mL, 48 h). (**D**) The effect of PP242 on the permeability of FITC-dextran in TNF-α treated Caco-2 cell monolayers. Data were shown as mean ± SD and replicated three times. ** *p* < 0.01, *** *p* < 0.001, **** *p* < 0.0001 versus control group. ## *p* < 0.01, ### *p* < 0.001 versus TNF-α group. n.s., no significant. Ctr, control group.

To investigate the effect of PP242 on increased claudin-2 expression caused by TNF-α, we used western blotting analysis and immunofluorescence staining to detect claudin-2 expression. Western blotting analysis showed that PP242 alleviated claudin-2 increase caused by TNF-α (Figure 5A). Consistent with this finding, immunofluorescence staining also demonstrated that PP242 rescued the enhancement of fluorescence induced by TNF-α (Figure 5B). In addition, we also investigated the change of intestinal barrier function by measuring TER and the permeability of FITC-dextran in TNF-α treated Caco-2 cell monolayers. We observed that the TER value obviously decreased and the dextran leakage from the apical to the basolateral compartment in the Transewell system significantly increased (Figure 5C,D). At the same time, we studied the role of PP242 in the change of barrier function induced by TNF-α, and found that PP242 partly rescued the TER change induced by TNF-α while it had no effect on the permeability (Figure 5C,D). Therefore, intestinal epithelial tight junction function was impaired after TNF-α treatment and PP242 partly rescued it.

### 2.6. Repeated Experiments in IEC-6 Cells Confirmed PP242 Alleviated the Change of Lysosome pH, Autophagic Flux, Claudin-2 Protein, and Intestinal Epithelial Tight Junction Function Induced by TNF-α

To further examine whether the results above extended to other intestinal cell, we repeated experiments above in IEC-6 cell monolayers. First, our experiments showed that TNF-α was significantly enhanced the expression of claudin-2, LC3B-II, and P62 (Figure S1A–C). Then we found that this increase of LC3B-II sustained when pretreated with 3-MA but disappeared when pretreated with bafilomycin A1 (Figure S1D,E). In ad-mCherry-GFP-LC3B transfected cells, more yellow LC3 puncta was observed in TNF-α treatment group compared to the control group (Figure S1F,G). In addition, both the qualitative and quantitative assay of lysosomal pH indicated that TNF-α increased the lysosome pH (Figure S2A,B). Last, our results demonstrated that the increase of lysosome pH, disruption of autophagy flux, elevation of claudin-2 level, and decease of TER induced by TNF-α were alleviated by the autophagy inducer PP242, which also promotes lysosome biogenesis (Figure S2B–F). All these data were similar to results of Caco-2 cell experiments, which further confirmed the role of autophagy in the change of claudin-2 and intestinal epithelial junction function induced by TNF-α.

## 3. Discussion

Inflammatory conditions, which are accompanied by intestinal tight junction function changes, are common in IBD and NEC [5,6]. Pro-inflammatory cytokines, especially TNF-α, act an important role in those tight junction function changes, and several studies have been proved that TNF-α antagonists provide benefit to patients or rat models [19,20]. However, the precise mechanism in this process is largely unknown. It has been shown that autophagy related genes are risk factors of IBD [21] and autophagy is also related with claudin-2 levels or intestinal tight junction function changes in starvation conditions [12]. In addition, autophagy status dynamically changes in different conditions [13,14]. Therefore, the aim of our study is to explore whether autophagy acts a role in the change of claudin-2 expression and intestinal epithelial tight junction function induced by TNF-α.

Autophagy is a dynamically catabolic process to maintain cellular homeostasis and provides energy. Studies have shown that autophagy changes when TNF-α administration, but conclusions are contradictory [22,23,24,25]. Unlike our results, some studies have suggested that TNF-α induces autophagy only according to the increase of LC3B-II levels [22,23]. However, autophagy is a catabolic pathway containing a series of process such as initiation, nucleation, elongation, fusion, and degradation, thus only evaluating the expression of LC3B-II is not large enough to explain the whole process of autophagy under certain administration. Therefore, our research has provided more evidence to correctly understand the real status of autophagy after TNF-α treatment. To clarify whether the increase of LC3B-II depends on the induction of autophagy or the inhibition of autophagic degradation, we applied P62 as another autophagy marker and found that TNF-α treatment dramatically increased its level, implying the inhibition of autophagy flux. In addition, we also used 3-MA and bafilomycin A1 for autophagy flux assay which further confirmed the disruption of autophagic degradation during TNF-α treatment. Finally, transfection of ad-mCherry-GFP-LC3B to cells before TNF-α treatment showed that the number of yellow dots were markedly increased while the red dots were not increased, also suggesting the process of autophagic degradation was blocked. All these results clearly indicated that the inhibition of autophagic degradation was responsible for the accumulation of LC3B-II induced by TNF-α.

In the process of autophagic degradation, the acidic condition of lysosome is vital for activation of lysosomal enzymes [17]. Wang et al. have shown that TNF-α increased the lysosomal pH in dopaminergic cells and this increase was attributed to the inhibition of autophagic degradation [24]. Similarly, our study also found the inhibition of autophagic degradation in TNF-α treated cells resulted from the alkalizing of lysosome. Moreover, mTOR inhibitor PP242, which also known as an activator of lysosome biogenesis [18], was proven to largely rescue the increase of pH and the inhibition of autophagy flux, which in turn clarified the role of lysosome pH in TNF-α treated cells. Overall, these results suggested that the alkalizing of lysosome may account for autophagy flux impairment in TNF-α treated cells.

Claudin-2, which belongs to the claudin family of proteins, has been shown to be regulated by a variety of pathways. In this research, we found claudin-2 increases after TNF-α administration which is consistent with those reported by Mankertz et al., who thought phosphoinositide 3-kinase (PI3K) pathway plays an important part in this process [4]. Furthermore, some studies demonstrated that extracellular signal-related kinases (ERK), Ras homolog gene family member A (RhoA), and epidermal growth factor receptor (EGFR) pathway have an impact on claudin-2 expression [26,27,28], and Yasaman et al. have proved that these pathways take part in TNF-α induced claudin-2 elevation by reducing claudin-2 degradation [29]. Similarly, Nighot et al. reported that claudin-2 is degraded by autophagy pathway during starvation condition [12]. In our experiments, we found that autophagy inhibitor 3-MA increased claudin-2 expression, implying claudin-2 may degraded by autophagy. Moreover, using PP242 to rescue autophagy flux inhibition induced by TNF-α also relieved the increase of claudin-2. All in all, these studies indicated that the inhibition of autophagic degradation process contributes to the elevation of claudin-2 protein after TNF-α treatment.

Previous study has demonstrated that claudin-2 enhances the intestinal permeability and decreases TER values [12,30]. Similarly, our study showed that the elevation of claudin-2 induced by TNF-α is accompanied with the increase of intestinal permeability and the reduction of TER value. However, PP242 mediated claudin-2 remission only partly alleviated the TER changes in TNF-α treated cells, but had no effects on intestinal permeability. This phenomenon may be due to the fact that the pore pathway consisted of caludin-2 and other protein is only accountable for the small molecule materials with a molecular radius of <4 Å [30,31], while the maker FITC-Dextran we used in this study is a large-sized probe. Given that Nighot et al. have demonstrated that only claudin-2 protein contributes to the change of TER during autophagy process [12], and thus we mainly focused on the claudin-2 protein in the current study. Our results revealed that intestinal epithelial tight junction dysfunction induced by TNF-α was partly attributed to the elevation of claudin-2, and our ongoing studies will further evaluate other tight junction proteins that may give a much more detailed insight into the underlying mechanisms.

TER is a parameter representing the ionic conductance of tight junctions, which mainly reflects the tight junction function [32]. Previous studies showed that occludin had minor effect on TER [33] while claudin-2 largely affected TER through the modulation of the ion channels [12]. The aberrant expression of claudin-2 has been found in many diseases such as IBD, Celiac disease, and HIV-enteropathy [34]. Moreover, the upregulation of claudin-2 may be related to diarrhea and early neoplastic transformation [35,36]. Thus, PP242, which was shown to protect against the change of TER induced by TNF-α, may have an important clinical significance in these diseases.

PP242, a dual mTOR complex 1 (mTORC1) and mTOR complex 2 (mTORC2) inhibitor, has been demonstrated to suppress proliferation and induce apoptosis in tumor cells [37,38]. Previous studies also found mTOR pathway is active in IBD and mTORC1 inhibitor Rapamycin is effective to refractory IBD patients [39]. In this study, we found that PP242 alleviates the claudin-2 increase and tight junction function impairment in TNF-α treated cell monolayers via autophagy-lysosome pathway, which may partly explain its therapeutic effect in IBD. Nevertheless, we should consider that the protective effect of PP242 may be attributed to other pathways (cell growth, proliferation, and survival, etc.) which can be modified by PP242. In addition, studies have shown that PP242 promoted lysosome biogenesis through phosphorylating transcription factor EB (TFEB) [18,40] and we speculate that TNF-α may inhibit autophagic degradation via TFEB signaling. Therefore, our research has emphasized the importance of the mTOR pathway in IBD and further studies are needed to address the involving pathways.

In conclusion, our study reveals that the accumulation of claudin-2 and epithelial tight junction function impairment is partly caused by the inhibition of autophagic degradation in TNF-α treated cell monolayers. This study provides new insight for the mechanism of claudin-2 increase in TNF-α treated cell monolayers and emphasizes the importance of autophagy in this process, which may promote our understanding of the etiology of inflammatory diseases. In addition, autophagy-lysosome-mediated degradation may be an important pathway to regulate tight junction function which is largely influenced by TJs.

## 4. Materials and Methods

### 4.1. Cell Culture and Reagents

Caco-2 cells and IEC-6 cells were obtained from the Cell Bank of the Chinese Academy of Sciences (analog no. TCHu146, Shanghai, China) and Shanghai Fuxiang Biotechnology (catalog no. CRL-1592, Shanghai, China) respectively. Cells were cultured in DMEM supplemented with 10% fetal bovine serum and 1% penicillin/streptomycin. Cells were maintained in a 5% CO_2_/95% air atmosphere at 37 °C. Recombinant TNF-α was obtained from R&D Systems (catalog no. 510-RT-010, Minneapolis, MN, USA). Bafilomycin A1 (catalog no. 189490) and 3-methyladenine (catalog no. 19-148) were purchased from EMD Millipore (Temecula, CA, USA). PP242 was purchased from Santa Cruz Biotechnology (catalog no. sc-301606, Santa Cruz, CA, USA). Claudin-2 antibody (catalog no. 51-6100, Invitrogen, Carlsbad, CA, USA), LC3B antibody (catalog no. L7543, Sigma, St. Louis, MO, USA), P62 antibody (catalog no. sc-25575, Sancta Cruz Biotechnology, Santa Cruz, CA, USA), and β-actin antibody (catalog no. 4970, Cell Signaling Technology, Danvers, MA, USA) were purchased from relevant manufacturers. Ad-mCherry-GFP-LC3B was purchased from Beyotime Biotechnology (catalog no. C3011, Shanghai, China).

### 4.2. Western Blot Analysis

To isolate cellular samples, cells were rinsed with cold PBS twice and lysed in RIPA buffer (25 mM Tris HCl PH 7.6, 150 mM NaCl, 1% NP-40, 1% sodium deoxycholate, 0.1% SDS; Thermo Fisher, Rockford, IL, USA) containing PMSF. Protein concentration was evaluated by the bicinchoninic acid method using a BCA Protein Assay Kit (23227, Thermo Fisher, Rockford, IL, USA). Equal amounts of total protein were loaded into SDS-polyacrylamide gels for electrophoresis and transferred to a PVDF membrane (Millipore, Temecula, CA, USA). The membrane was blocked in 5% bovine serum albumin (BSA) in PBST for 2 h at room temperature before being incubated overnight at 4 °C with those primary antibodies: β-actin, Claudin-2, LC3B, and P62 antibody. After being reacted with secondary antibody, the membrane was detected with a ChemiDoc XRS^+^ system (Bio-Rad, Hercules, CA, USA). The density of specific protein bands was quantified by Image J software (version 1.49v, National Institute of Health, Bethesda, MD, USA).

### 4.3. Ad-mCherry-GFP-LC3B Transfection

Cells were grown on 24-well plates and reached 20%–30% confluence at the time of transfection. After two washes, cells were transfected with Ad-mCherry-GFP-LC3B adenovirus at a MOI of 80 in 200 μL DMEM containing 10% FBS for 24 h at 37 °C. Following indicated treatment, autophagy was observed under laser scanning confocal microscope (Leica, Wetzlar, Germany). Autophagy flux was evaluated by calculating the number of yellow and red puncta.

### 4.4. Lysosomal pH Measurement 

Lysotracker Red DND-99 (L7528, Life Technologies, Carlsbad, CA, USA) was used for qualitative analysis of Lysosomal pH. Previous study has showed that the red dye becomes more fluorescent in acidic environment while less in alkaline conditions [24]. After desired treatment, cells were added with 50 nM Lysotracker Red DND-99 in medium containing 10% FBS for 40 min at 37 °C in dark. After three washes, cells were fixed with 4% polyoxymethylene for 15 min. After another three washes, cells were incubated with DAPI nuclear stain solution for 5 min in dark and viewed under inverted fluorescence microscopy.

To quantify lysosomal pH, the method was detailed as previously described [41,42,43]. In brief, cells grown on the 96-well plate were labeled with 5 μm Lysosensor Yellow/Blue DND-160 (L7545, Life technologies) in regular medium for 5 min at 37 °C in dark. After being washed with PBS twice, cells were treated for 10 min with 10 μm monensin and 20 μm nigericin dissolved in 20 mM MES buffer (pH 3–6.5) containing 110 mM KCl and 20 mM NaCl. The fluorescence was measured by fluorescent microplate reader with emission wavelengths at 535 nm in response to excitation at 340 and 380 nm. The pH calibration curve of this fluorescence probe was performed with Graphpad Prism software (version 6.0c, GraphPad Software, La Jolla, CA, USA) according to the relationship between the ratio of excitation at 340 nm/380 nm and the pH value of each MES buffer. The lysosomal pH value of each sample was estimated using the calibration curve of this probe.

### 4.5. Immunofluorescence Staining

Cell monolayers grown on 24-well Transwell chambers (3470, Corning, Inc., Corning, NY, USA) were washed twice with cold PBS and fixed with 4% Polyoxymethylene for 15 min at room temperature. Then the cell monolayer was incubated with 3% BSA for 30 min at room temperature and labeled with claudin-2 antibody overnight at 4 °C. After being washed with PBS, cell monolayers were incubated with Alexa Fluor 488 AffiniPure Goat Anti-Rabbit IgG for 1 h at 37 °C. After two more washes, the cell monolayer was incubated with DAPI nuclear stain solution for 10 min. Fluorescence images were captured by inverted fluorescence microscopy (Leica).

### 4.6. Measurement of TER and Permeability

All experiments were carried out as previously reported [43,44,45]. In order to calculate TER values of cell monolayer, we used a Millipore electric resistance system (ERS-2; Millipore). Caco-2 cells or IEC-6 cells were seeded on permeable filters (0.4 nm pore size) in 24-well Transwell chambers (Corning, Inc., 3470) with a density of 2 × 10^5^ cells/mL for up to 14 or 6–7 days to form a cell monolayer, respectively. To evaluate the TER of each cell monolayer, the mean resistance value of unseeded Transwell inserts was subtracted from each value, and corrected for the area of the filter (0.33 cm^2^).

Permeability studies were performed with FITC-Dextran (FD-40, Sigma, St. Louis, MO, USA). Cells were grown on the filters in a 24-well Transwell chamber. After drug treatment, the media in the basolateral and apical compartment of the Transwell chamber was replaced with 0.6 mL DMEM or 0.1 mL DMEM containing FITC-Dextran at 1 mg/mL. After 1 h incubation, medium from the basolateral compartment was used for spectrofluorometric measurement of Dextran concentration.

### 4.7. Statistical Analysis

All data are shown as mean ± SD of at least triplicate experiments. Statistical difference was estimated using Student’s unpaired *t*-test (two-tailed) for two group comparison or one-way ANOVA with Tukey’s Multiple Comparison test for more than two groups by GraphPad Prism 6.0 software. *p* < 0.05 was set as statistically significant.

## 5. Conclusions

Our study reveals that TNF-α-induced claudin-2 increase and impairment of epithelial tight junction function are partly attributed to the inhibition of autophagic degradation in vitro. Further research is needed to evaluate other tight junctions that may play a role in this process. This research may provide a new insight for our understanding on the etiology of some inflammatory diseases.

## Supplementary Materials

Supplementary materials can be found at www.mdpi.com/1422-0067/18/1/157/s1.
