# Supplementary material for: Inhibition of Autophagic Degradation Process Contributes to Claudin-2 Expression Increase and Epithelial Tight Junction Dysfunction in TNF-α Treated Cell Monolayers
Source: Int J Mol Sci. 2017 Jan 17;18(1):157. doi: 10.3390/ijms18010157 (PMC5297790; doi:10.3390/ijms18010157)
Supplement: Supplementary file 1 [file ijms-18-00157-s001.pdf]

Supplementary Materials: Inhibition of Autophagic Degradation Process Contributes to Claudin-2 Expression Increase and Epithelial Tight Junction Dysfunction in TNF-α Treated Cell Monolayers

Cong Zhang, Junkai Yan, Yongtao Xiao, Yujie Shen, Jiazheng Wang, Wensong Ge and Yingwei Chen

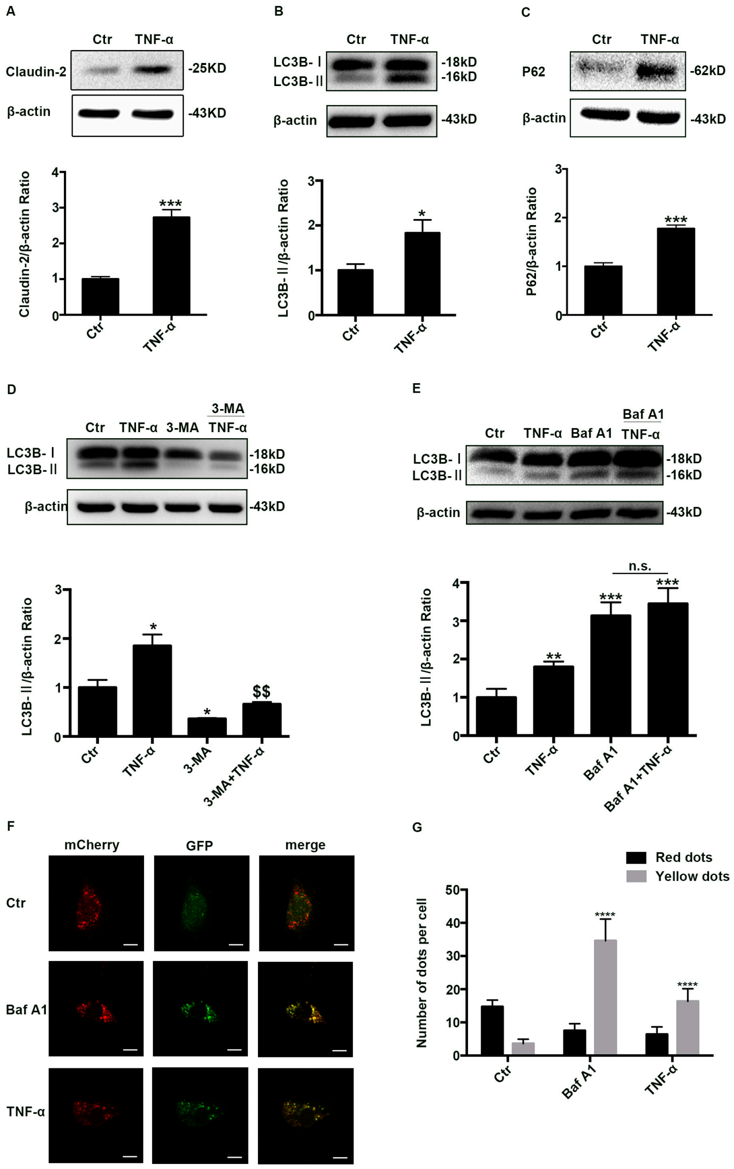

Figure S1. Claudin-2 increase and impaired autophagic flux in TNF-α treated IEC-6 cell monolayers. (**A**–**C**) Western blotting analysis of caludin-2, LC3B-II and P62 protein in IEC-6 cells after TNF-α (20 ng/mL, 48 h) treatment. (**D**,**E**) Western blot analysis of LC3B-II in cells treated with TNF-α (20 ng/mL) in the presence or absence of 3-MA (5 mM) or Baf A1 (100 nM) pretreatment. (**F**) Representative images of IEC-6 cells transfected with ad-mCherry-GFP-LC3B adenovirus after TNF-α (20 ng/mL) or Baf A1 treatment (100 nM). Scale bar: 10 μm. (**G**) The number of red and yellow LC3 dots per cell were counted under confocal microscope (>30 cells/group). Data were shown as mean ± SD and replicated in 3 times. * $p < 0.05$, ** $p < 0.01$, *** $p < 0.001$, **** $p < 0.0001$ versus control group. $$ $p < 0.01$ versus 3-MA group. n.s., no significant versus TNF-α group. Ctr, control group; 3-MA, 3-methyladenine; Baf A1, Bafilomycin A1.

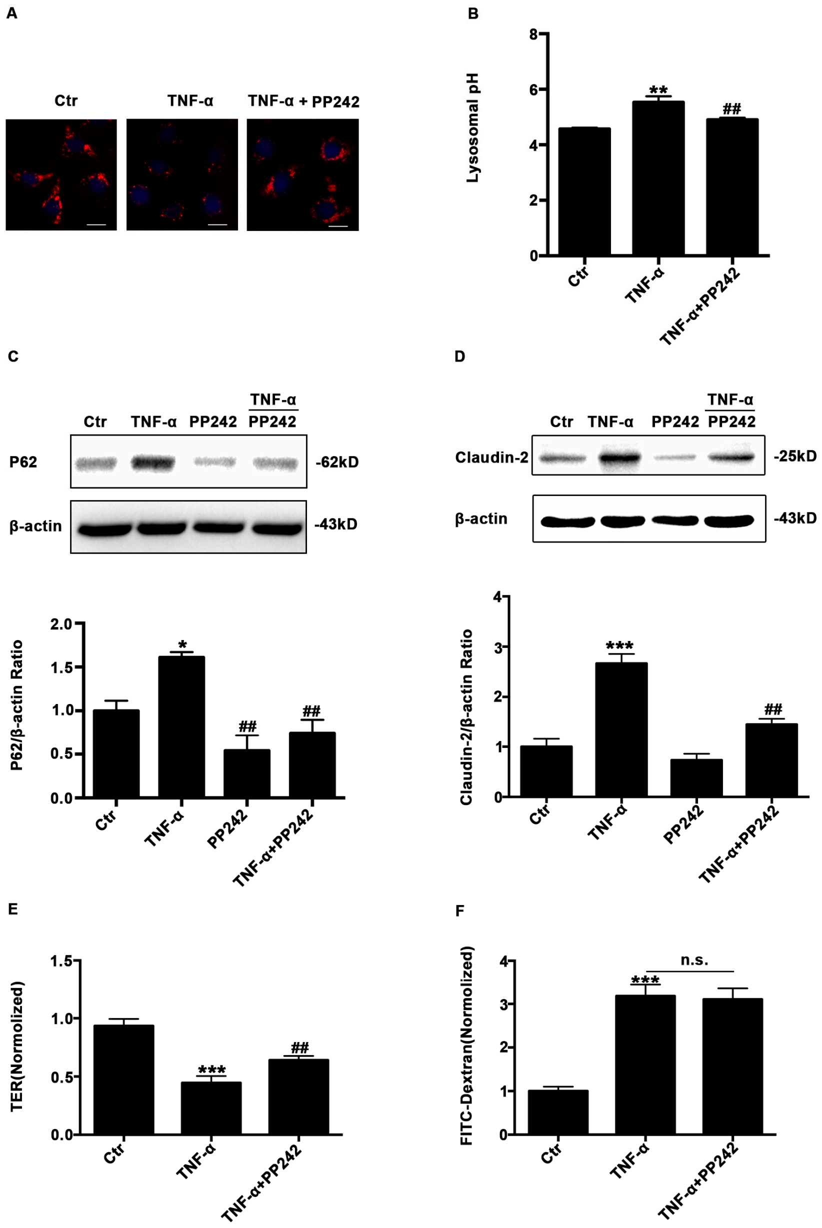

Figure S2. The effect of PP242 on the change of lysosomal pH, autophagic flux, claudin-2 and intestinal barrier function induced by TNF-*α* in IEC-6 cell monolayers. (**A**) Images of IEC-6 cells labeled with Lysotracker Red DND-99 dye after TNF-*α* (20 ng/mL, 48 h) and/or PP242 (1 μM, 24 h) treatment. Scale bar: 15 μm. (**B**) Lysosomal pH changes of IEC-6 cells labeled with Lysosensor Yellow/Blue DND-160 after TNF-*α* and/or PP242 treatment. (**C**,**D**) Western blot analysis of P62 and claudin-2 protein during TNF-*α* and/or PP242 treatment. (**E**,**F**) The effect of PP242 in TER and the permeability of the FITC-dextran in TNF-*α* treated IEC-6 cell monolayers. Data were shown as mean ± SD and replicated in 3 times. * $p < 0.05$, ** $p < 0.01$, *** $p < 0.001$ versus control group. ## $p < 0.01$ versus TNF-*α* group. n.s., no significant versus TNF-*α* group. Ctr, control group.
